# Alginate Particles as Platform for Drug Delivery by the Oral Route: State-of-the-Art

**DOI:** 10.1155/2014/926157

**Published:** 2014-04-09

**Authors:** Alejandro Sosnik

**Affiliations:** Group of Pharmaceutical Nanomaterials Science, Department of Materials Science and Engineering, Technion-Israel Institute of Technology De-Jur Building, Office 607, Technion City, 32000 Haifa, Israel

## Abstract

Pharmaceutical research and development aims to design products with ensured safety, quality, and efficacy to treat disease. To make the process more rational, coherent, efficient, and cost-effective, the field of Pharmaceutical Materials Science has emerged as the systematic study of the physicochemical properties and behavior of materials of pharmaceutical interest in relation to product performance. The oral route is the most patient preferred for drug administration. The presence of a mucus layer that covers the entire gastrointestinal tract has been exploited to expand the use of the oral route by developing a mucoadhesive drug delivery system that showed a prolonged residence time. Alginic acid and sodium and potassium alginates have emerged as one of the most extensively explored mucoadhesive biomaterials owing to very good cytocompatibility and biocompatibility, biodegradation, sol-gel transition properties, and chemical versatility that make possible further modifications to tailor their properties. The present review overviews the most relevant applications of alginate microparticles and nanoparticles for drug administration by the oral route and discusses the perspectives of this biomaterial in the future.

## 1. Introduction

Pharmaceutical research and development aims to design products with ensured safety, quality, and efficacy to treat disease. To make the process more rational, coherent, efficient, and cost-effective, the field of Pharmaceutical Materials Science (PMS) has emerged as the systematic study of the physicochemical properties and behavior of materials of pharmaceutical interest (MPIs) in relation to product performance [1]. MPIs are classified into two major groups, active pharmaceutical ingredients (drugs) and nonpharmacologically active pharmaceutical excipients. To systematize this study, PMS adopted the fundamentals of the materials science tetrahedron (MST): a deep understanding of the structure-properties relationship and the study of processing methods to achieve the expected (bio)pharmaceutic performance* in vitro* (e.g., mechanical strength, compressibility, physicochemical stability) and* in vivo* (e.g., bioavailability) [2, 3]. The most common (bio)pharmaceutic drawback that pharmaceutical scientists face is poor aqueous solubility [4–7], followed by limited drug permeability [4, 7] and physicochemical instability [8]. All of them, in one way or another, challenge early stages of drug screening and formulation and decrease oral bioavailability. Adverse effect due to systemic exposure represents another main limitation of the pharmacotherapy [9]. For instance, it is estimated that toxicity is responsible for the attrition of one third of the new drug candidates. In this framework, different micro- and nanotechnologies have been implemented to enhance the different (bio)pharmaceutic aspects of drugs and to increase the chances of translation into clinics [10, 11].

Oral administration (p.o.) is the most extensively used [12, 13]. Regretfully, p.o. entails a variety of hurdles such as incomplete absorption, degradation in the gastrointestinal tract, hepatic metabolism, and limited bioavailability. In addition, since the gastrointestinal residence time of conventional formulations is relatively short (a few hours), this route is less optimal for sustained release purposes [14].

Mucus is a complex combination of glycoproteins [15] that covers the different segments of the gastrointestinal tract with varying thicknesses and compositions [16] thus conferring the mucus well defined rheology, film formation capacity and adhesiveness [15]. These anatomical features have been exploited to expand the use of the oral route. In this context, a broad spectrum of pharmaceutical products that incorporate natural, synthetic, and semisynthetic mucoadhesive excipients have been developed. These excipients, apart from being pharmacologically inert, are expected to establish an intimate interaction with the gastrointestinal mucus and to govern the performance of the drug delivery system upon administration. Since the emergence of microtechnology and nanotechnology, breakthrough progresses have been made in the development of innovative mucoadhesive products for immunization, diagnosis, and treatment of disease [17–20].

**Figure 1:**
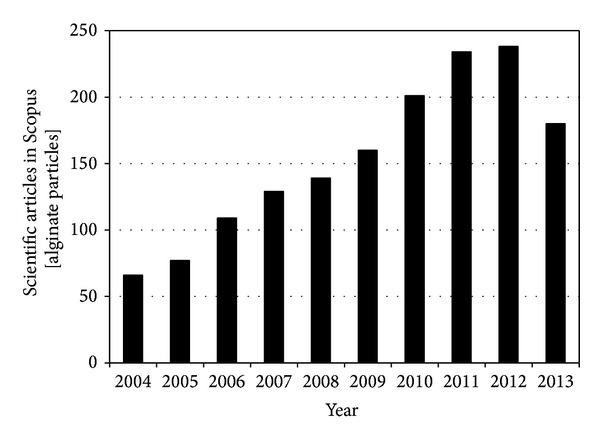
Progression of the number of scientific articles in the search engine Scopus for alginate particles over the last decade.

Alginic acid and sodium and potassium alginates (ALG) have emerged as one of the most extensively explored mucoadhesive biomaterials owing to very good cytocompatibility and biocompatibility, biodegradation [21], sol-gel transition properties, and chemical versatility that make possible further modifications to tailor its properties [22]. The interest of the scientific community in ALG as a platform for the development of micro and nanodrug delivery systems has given place to a steady growth of the available literature over the last decade (Figure 1). This also found an expression in a rich and growing intellectual property [23]. The present spotlight paper reviews the most relevant applications of ALG microparticles and nanoparticles for drug administration by the oral route.

## 2. Structure and Properties of ALG 

**Figure 2:**
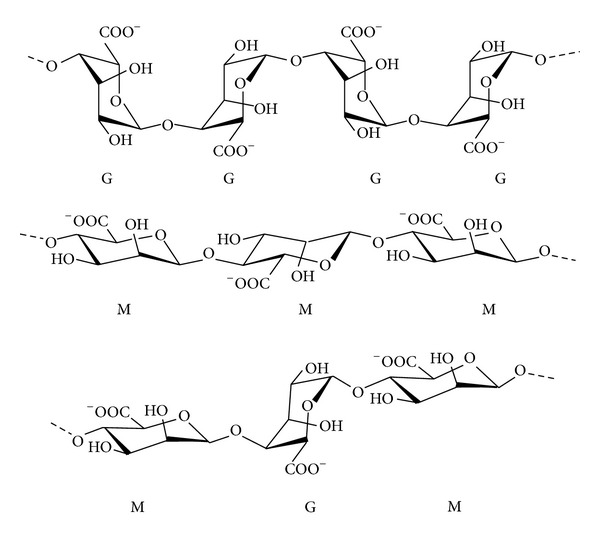
Structure of ALG. Reproduced from [21] with permission of Elsevier.

**Figure 3:**
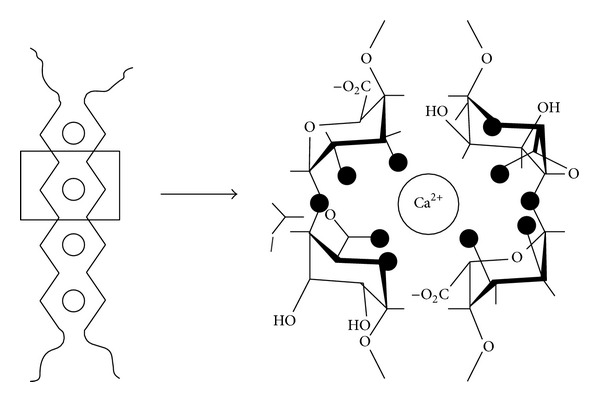
Schematic drawing and calcium coordination of the “egg-box” model, as described for the pair of guluronate chains in calcium ALG junction ones. Dark circles represent the oxygen atoms involved in the coordination of the calcium ion. Reproduced from [31] with the permission of the American Chemical Society.

ALG is a generic name assigned to a series of natural unbranched polyanionic polysaccharides of *β*-D-mannuronic acid (M) and *α*-L-guluronic acid (G) repeating units linked by a 1→4 linkage and displaying chain homosequences of MMMMM and GGGGG, interdispersed with MGMGMG heterosequences (Figure 2) [21]. ALG is isolated from a variety of mainly brown algae such as* Macrocystis pyrifera*,* Laminaria hyperborea,* and* Ascophyllum nodosum* [24–26]. Molecular weights in the 32 to 400 kg/mol range together with different relative G/M compositions and chain arrangements based on the source of extraction and the age of the algae [24, 27, 28] resulted in the commercialization of over 200 ALG types [29]. In addition, the rheological and drug delivery performance of ALG is also conditioned by the G/M ratio, the molecular weight, the concentration, and the pH of the medium. One of the most appealing features of ALG is the ability to crosslink water solutions ionotropically by a mechanism whereby pendant carboxylic acid moieties of G units chelate Ca^2+^ and other divalent cations (e.g., Sr^2+^, Ba^2+^) to generate 3D networks [29, 30]. This gelation mechanism is explained by the “egg-box” model where one divalent cation interacts with four –COOH groups (Figure 3) [31] and it has been exploited over more than three decades for the encapsulation of a broad spectrum of drugs [30, 32], proteins [33, 34], genes [35, 36], and cells [21, 37–40]. Another cation that was used to crosslink ALG is Fe(III) [41].

The mechanical and the physical stability of ALG gels depends on the G content, the greater the G content, the more rigid and brittle the matrix. The process can also be reverted in the presence of ion sequestrants such as ethylenediaminetetraacetic acid (EDTA) [42] or sodium citrate [43]. Also, ALG gels tend to be eroded under more neutral and basic pH values than under acid conditions [44]. This property has motivated its use in the chemical stabilization of drugs and biologicals of oral administration that are not stable in gastric fluids.

From a regulatory point of view, the U.S. Food and Drug Administration (US-FDA) recognizes ALG as a “Generally Referred As Safe” (GRAS) material, a designation that applies to substances accepted as safe for alimentary use by qualified experts [45]. GRAS excipients are listed in the Code of Federal Regulations Title 21 (21 CFR) parts 182 and 184 [46]. This status is very advantageous at the time of the development of products with good chances of bench-to-bedside translation. In addition, ALG is bioadhesive and mucoadhesive, biocompatible and nonirritant, thus finding application in the production of adhesive tablets for buccal drug delivery [47] and wound dressings with different features such as exudates absorption, moisture conservation, and wound healing [48–51]. The prothrombotic activity of ALG has also resulted in its use as haemostat [52, 53]. At the same time, there is still some controversy regarding specific adverse effects such as immunogenicity [54, 55] that would most likely stem from the quality and the level of purity of the biomaterial employed in the different studies [21] and from an intrinsic immunogenic nature, for example, traces of heavy metals, endotoxins, proteins, and polyphenolic compounds that could remain in the final product and lead to undesired effects. When purified in a multistep extraction methodology, no foreign body response was apparent in animals upon intramuscular implantation [38, 56]. These findings supported the hypothesis that adverse effects would stem from impurities rather than from the intrinsic immunogenicity of the biomaterial. Moreover, owing to its gelation capacity, ALG has been exploited in cell encapsulation and immunoisolation [39, 40, 57, 58], endovascular embolization [59], and depot drug delivery [60]. Studies of this kind are very relevant because they would support the use of ALG by different parenteral routes (e.g., intra-articular). At the same time, it should be stressed that ALG has not been approved yet for parenteral administration. The development of an ultrapurified low endotoxin ALG could pave the way to applications with stricter biocompatibility demands [61].

Due to the hydrophilic nature of ALG, the release of encapsulated drug payloads could follow different mechanisms. Water-soluble drugs are mainly released by diffusion, while poorly water soluble drugs by matrix erosion. The release of small molecules is fast due to the fact that swelled ALG matrices display a pore diameter of approximately 5 nm [54]. However, different modifications can be pursued to physically or chemically bind the drug to the network and to prolong the release. An additional interesting feature of ALG is that dry systems are mucoadhesive, prolonging the residence time and the release in different mucosal tissues such as intestine, lung, nose, and eye [62–67]. Due to the high chemical functionality (two –OH and one –COOH per repeating unit), the chemical modification of the side chain of ALG has been extensively explored to increase its solubility in aprotic solvents [27, 68], to modify other physicochemical properties [69, 70], to attain biomimetic [71] and amphiphilic features [72], and to conjugate cell signalling ligands [73, 74]. For an extensive and thorough overview of the chemical modification of ALG, the readers are referred to the review of Pawar and Edgar [22].

## 3. Alginate Applications in Oral Drug Delivery 

ALG has been employed for the production of a broad spectrum of drug delivery systems. The present paper will be focused on microparticles and nanoparticles because they represent the most innovative and promising developments [32].

### 3.1. Alginate Microparticles

**Figure 4:**
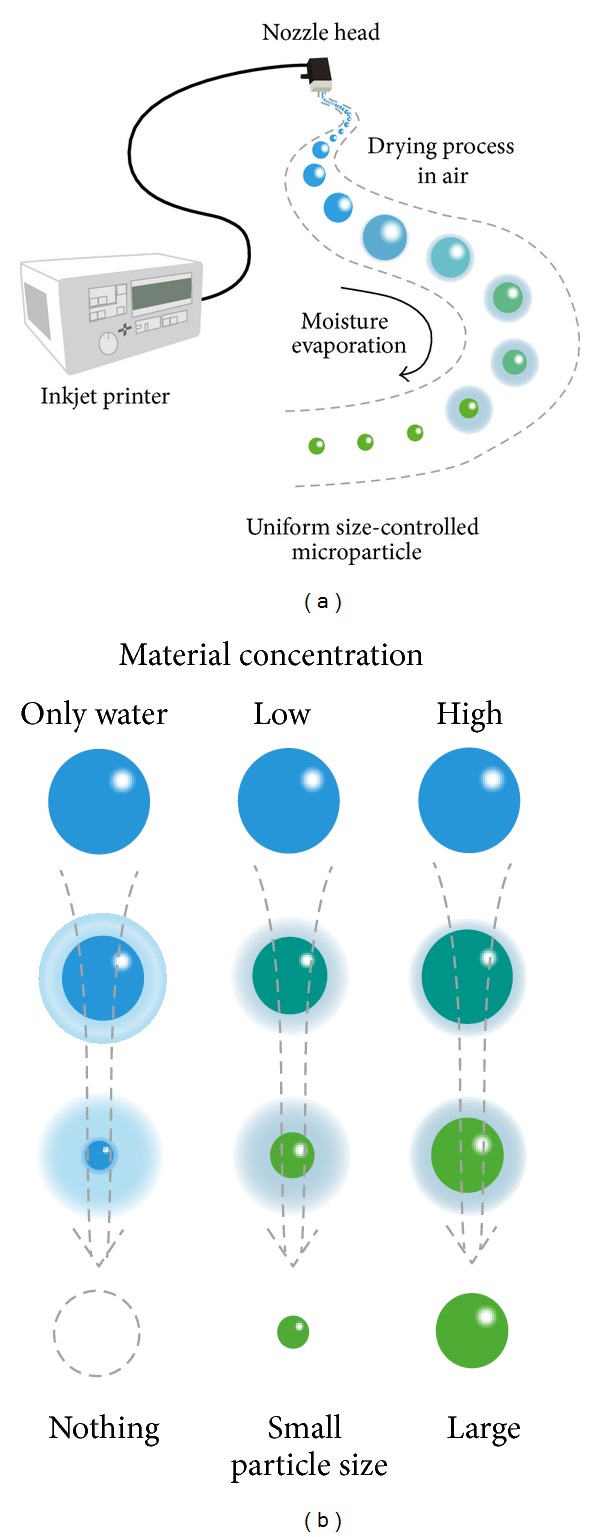
Concept for the production of uniform size-controlled microparticles with inkjet printer. (a) Microparticles are easily fabricated by evaporating moisture in air, and (b) size of particles could be controlled with changing the concentration of the biomaterial. Reproduced from [86] with permission of Elsevier.

**Figure 5:**
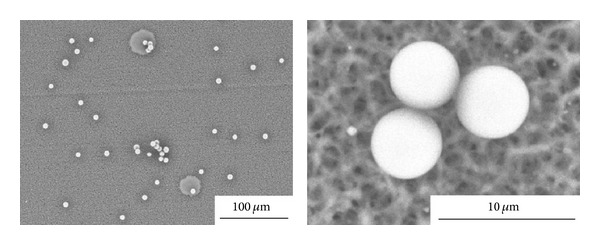
SEM micrographs of ALG microparticles fabricated by inkjet/drying using a 0.8% ALG solution. Reproduced from [86] with permission of Elsevier.

ALG microparticles (<200 *μ*m) can be produced by different techniques [75] such as air atomization [75–77], emulsification [78, 79], and complexation with counterion polymers [80, 81], often combined with additional technologies. More innovative methods (e.g., spray-drying [82], electrohydrodynamic atomization [83], impinging aerosols [84, 85], and inkjet/drying process [86]) that enable a better control of the size and size distribution have been also reported. For example, Iwanaga et al. developed an inkjet device for the fabrication of microparticles with an unprecedented size control (Figure 4) [86]. The precise adjustment of the parameters led to very low size distributions, optimal for scale up purposes in pharmaceutical development (Figure 5) [86].

**Figure 6:**
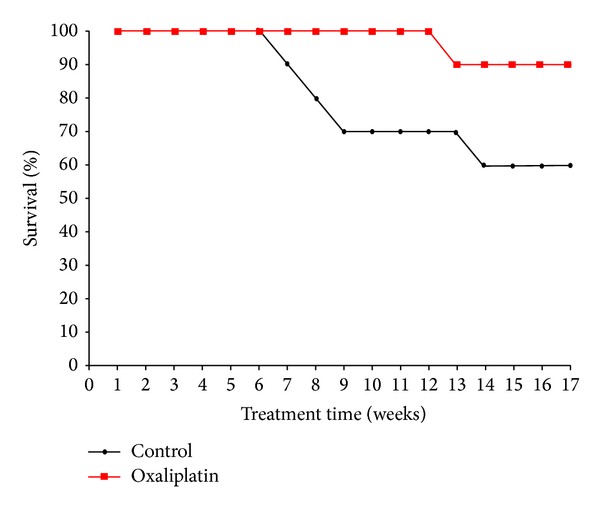
Animal survival profile over 17 weeks. Control group animals gavaged with drug-free ALG-chitosan microcapsules suspended in PBS and treatment group animals gavaged with microencapsulated oxaliplatin nanoparticles suspended in PBS. Reproduced from [91] with permission of Elsevier.

**Figure 7:**
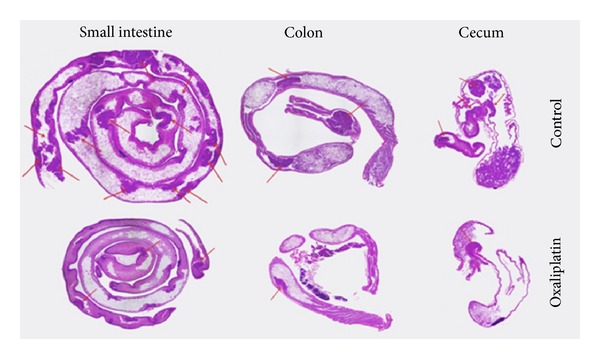
Hematoxylin and eosin tissue staining of mice polyps in small intestine, colon, and cecum for control and oxaliplatin treatment groups. Control mice had a higher number of polyps, indicated with red arrows, in the small intestine, colon, and cecum compared with oxaliplatin treatment group. Reproduced from [91] with permission of Elsevier.

**Figure 8:**
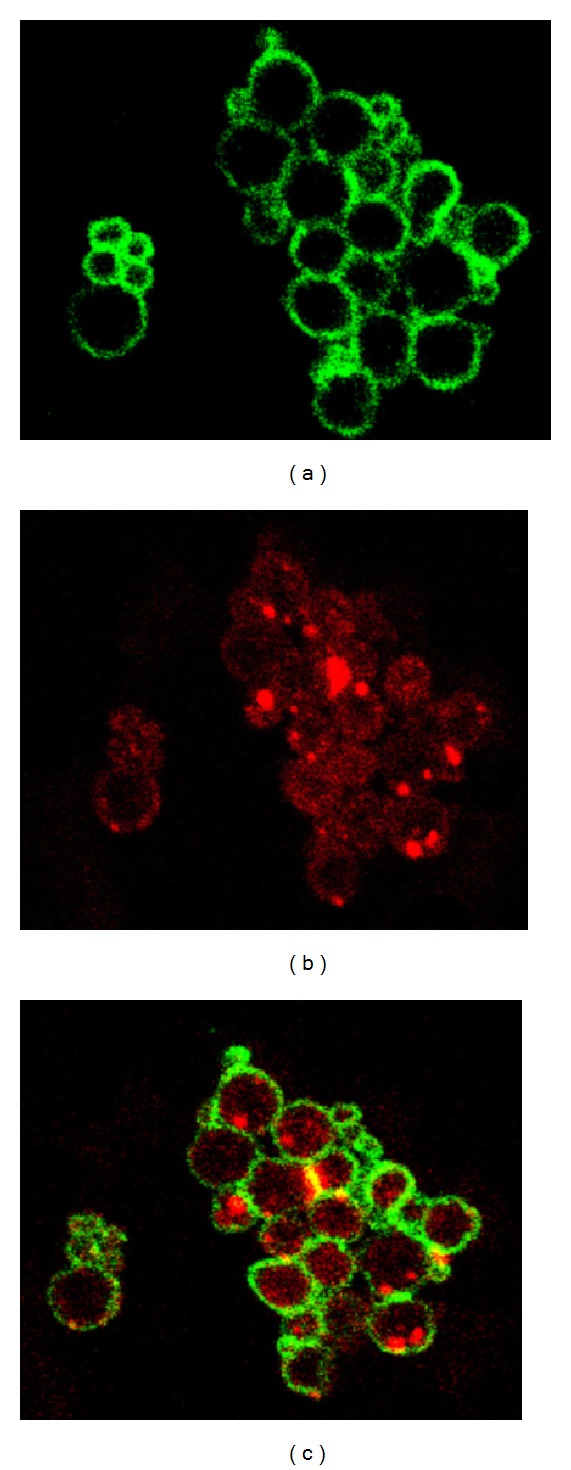
Confocal laser scanning microscopy of 5-aminosalycilic acid-loaded chitosan-Ca-ALG microparticles. (a) Fluorescein isothiocyanate-labeled chitosan (green), (b) rhodamine isothiocyanate-labeled ALG (red), and (c) image obtained by superposition. Reproduced from [93] with permission of Elsevier.

Other methods that provide very good control of the size are electrostatic droplets combined with external ionotropic gelation [87] and nebulization/gelation [88]. These technologies have been used for the encapsulation of cells, drugs, and proteins owing to the mild conditions required. A main disadvantage of calcium-crosslinked ALG is low mechanical stability that results in fast drug release. Thus, ALG microparticles are usually coated with polycationic polymers such as chitosan and poly-L-lysine that reduce swelling and increase their mechanical integrity in biorelevant media [89–91]. As mentioned above, the use of ALG for the systemic and localized delivery by the oral route has increased over the years. In this framework, intestinal and colonic release emerged as the most intensively investigated ones. For example, Coppi et al. produced ALG microparticles of diameter smaller than 3 *μ*m by spray-drying for uptake of M cells of the Peyer's patches and the targeting of polymixin B to the Gut Associated Lymphoid Tissue [92]. Particles were crosslinked with chitosan and calcium and they were gastroresistant. More recently, Urbanska et al. encapsulated oxaliplatin, a slightly soluble antitumoral drug, within chitosan-coated ALG microparticles for oral administration in colorectal cancer [91]. This formulation significantly prolonged the survival of C57BL/6J-Apc^*Min*⁡/+^ mice with respect to blank microparticles (Figure 6). The study was complemented with histopathology, where the different gastrointestinal compartments were stained with hematoxylin and eosin. Control groups showed tubular adenomas that protruded into the colon lumen and polypoid adenomas in the small intestine, while the oxaliplatin groups showed microadenomas (Figure 7) [91]. Surprisingly, the effect of free oxaliplatin nanoparticles was not assessed. This animal subset would have been of relevance to confirm whether the prolonged survival exclusively relied on the drug or, conversely, on the mucoadhesive drug delivery system employed for its administration and localized release. Within the same conceptual framework, Mladenovska and collaborators have developed ALG microparticles loaded with 5-aminosalicylic acid that were crosslinked and coated with calcium and chitosan employing a spray-drying technique for application in inflammatory bowel disease [93]. An interesting aspect of this study was the confirmation that the drug undergoes amorphization during the production process. This is critical to ensure its fast dissolution in the intestinal tract. Also, by using fluorescently labeled ALG and chitosan, the distribution of both polysaccharide components in the microparticles was elegantly shown (Figure 8) [93]. These results confirmed that the chitosan coating was restricted to the surface of the microparticle and it did not penetrate the matrix. ALG microspheres prepared by the emulsification technique have been also assessed for the encapsulation of more complex and sensitive payloads such as the tetanus toxoid intended for mucosal immunization [94]. The size of the microparticles was 1.34 *μ*m with very smooth surface and low surface porosity. The encapsulation efficiency reached almost 50% and the burst effect was relatively low. Furthermore, results showed that the antigenicity was conserved in 91% with respect to the free toxoid.

Another strategy to improve the mechanical stability of ALG matrices in aqueous media is by blending it with polycationic polymers (e.g., chitosan, pectin, gelatin) to form polyelectrolyte complexes. Jaya et al. encapsulated aspirin within ALG/pectin microspheres (90 *μ*m) by means of a homogenization/atomization and calcium crosslinking method and assessed the effect of the composition on the release kinetics [95]. The release was slow and controlled in the pH range between 1.2 and 8.2, what covers the conditions of the gastrointestinal tract. In addition, an increase of the pectin content increased the release rate. These results shed light on the versatility of ALG and the ability to fine tune the (bio)pharmaceutic performance by changing production methods and qualitative and quantitative compositions.

Encapsulation can be also used to prevent noxious effects of the encapsulated drug on the gastrointestinal mucosa, such as the ulcerogenicity of diclofenac sodium and other nonsteroidal anti-inflammatory drugs [96].

**Figure 9:**
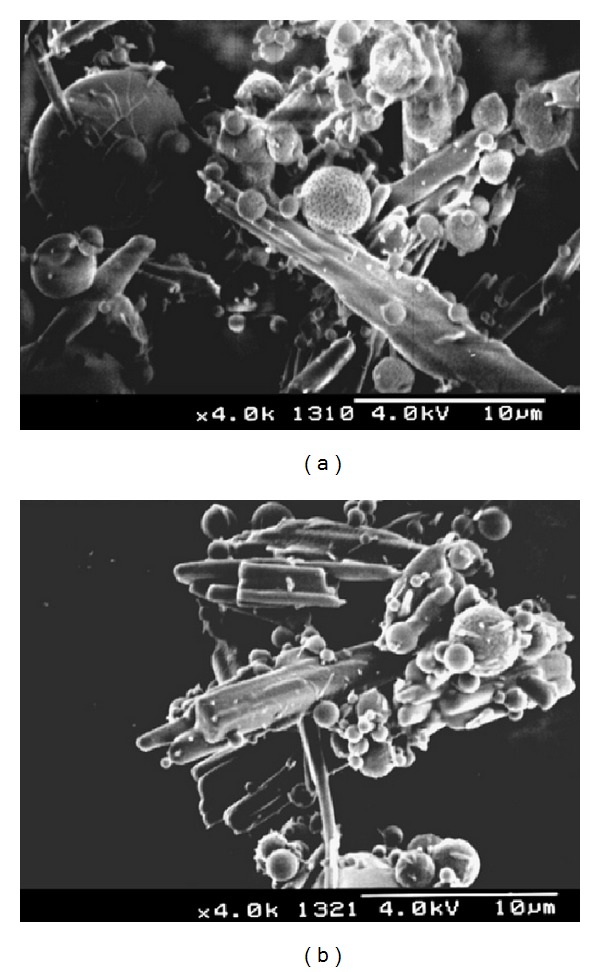
SEM micrograph of tandolapril-loaded ALG microparticles. The drug payload was 50% of the dry weight. (a) Microparticles without lactose and (b) microparticles with lactose. Scale bar = 10 *μ*m. Reproduced from [98] with permission of Elsevier.

Another method to produce drug-loaded ALG microparticles is to use drug microcrystals as template to produce polyelectrolyte multilayers or to directly coat them with ALG [97, 98]. In the case of multilayered systems, ALG is combined with a polycationic polymer and both layers are deposited following an alternated pattern [97]. An advantage of this technique is the very fine control of the capsule thickness, the microparticle diameter, and the release rate. At the same time, it should be pointed out that this approach is more laborious than more conventional ones where the properties of the final system can be adjusted by changing parameters such as drug/polymer ratio, molecular weight of the polymeric components, the concentration of the counterionic polymer and calcium ions in the hardening medium, and the hardening time [99]. In fact, multilayered coating appears as less feasible to scale up and translate into clinics than common processing methods. This is the reason why most of the works were devoted to modify and optimize standard technologies rather than explore more sophisticated and less reproducible ones. In this framework, Makai et al. used spray-drying for coating pure trandolapril microparticles with ALG, results being acceptable [98]. However, part of the drug remained nonencapsulated, as apparent from SEM analysis (Figure 9) [98]. This is a very common phenomenon when the interaction between the drug and the matrix is not sufficiently strong or when the morphology of the drug particles is nonspherical (e.g., needle-like) and they disrupt the microparticle surface. To overcome this disadvantage, particles could be film-coated with polymers that provide an integral outer surface layer. Aiming to improve the transport across the intestinal epithelium of the water-soluble antibiotic gentamicin, Iannuccelli et al. used a different approach [100]. The cationic drug was initially complexed with the polyanionic ALG, and then ALG was crosslinked with chitosan and calcium ions. Microparticles were translocated via M cells and follicle associated epithelium of the Peyer's patches and enterocytes, as demonstrated in* ex vivo* perfusion assays with rabbit and rat intestine containing Peyer's patches and* in vitro* in a Caco-2 cell monolayer model. However, only M cells transported the microparticles to subepithelial regions. This is a very interesting result and suggests the possible saturation of this transport pathway. The same group explored a similar drug delivery system to target the antitumoral tamoxifen to the lymphatic system [101].

The fast release of the encapsulated drugs is a relevant drawback of ALG. To stabilize the matrix and sustain the release, microparticles could be also obtained by forming interpenetrated polymer networks of ALG with other polysaccharides and natural polymers that are crosslinked ionotropically and/or covalently with different coupling agents [63, 102–104]. The later stage enables a better control of the matrix porosity and swelling and mechanical properties and consequently of the release rate. In this framework, Kulkarni and coworkers developed interpenetrating network beads made of ALG, gelatin and egg albumin that were crosslinked with glutaraldehyde to increase the half-life of the antibiotic cefadroxil [105]. The encapsulation efficiency was as high as 88% and the burst release was relatively low. Moreover, the release was sustained for at least 7 h.

ALG blends with cellulose derivatives, poly(acrylate)s, and pristine and modified polysaccharides have been also investigated. For example, Babu et al. prepared ALG/methylcellulose spherical microparticles by a water-in-oil emulsion method, crosslinked them with glutaraldehyde, and loaded them with nifedipine [106]. The drug was dispersed at the molecular level and the release was controlled for over 12 h. In another kind of application, the same research group used a similar system for the release of a organophosphate insecticide [107]. Angadi et al. produced blend microbeads of ALG and sodium carboxymethyl cellulose and coated them with chitosan for the controlled release of amoxicillin in the stomach to treat the infection by* Helicobacter pylori* [108]. The size was between 745 and 889 *μ*m, the upper limit for a microparticle, though this example is described because it addressed gastric release as opposed to a majority of works that focus on the intestinal one. In addition, the encapsulation efficiency was in the 52–92% range. The coating reduced the burst effect and sustained the release over more than 8 h under gastric-like pH conditions following an anomalous release kinetics. A limitation of this work is that it did not report on the drug concentrations attained and whether they were or were not within the therapeutic concentration. Mennini et al. developed ALG/chitosan microspheres for colonic delivery of celecoxib-*β*-cyclodextrin-polyvinylpyrrolidone complex in two kinds of therapy: (i) systemic in chronotherapic treatment of arthritis and (ii) local in prophylaxis of colon carcinogenesis [109]. Another inhibitor of the cyclooxygenase-2 enzyme, valdecoxib, was also encapsulated within Eudragit S100 and sodium alginate microparticles for colonic release [110]. More recently, pH- and thermoresponsive microspheres of ALG and poly(*N*-isopropylacrylamide)-*g*-guar gum were obtained by emulsion coupled to chemical crosslinking with glutaraldehyde to encapsulate the antituberculosis drug isoniazid [111]. The release was sustained for at least 12 h with a strong dependence on the pH of the medium; the release increased at pH 7.4 with respect to 1.2. Furthermore, the incorporation of graft copolymer enabled a much better control of the release kinetics with a substantial decrease of the burst effect. The modulation of the release using external stimuli (e.g., electrical current) has also been explored, though not in the case of particles but of macroscopic hydrogels [112].

**Figure 10:**
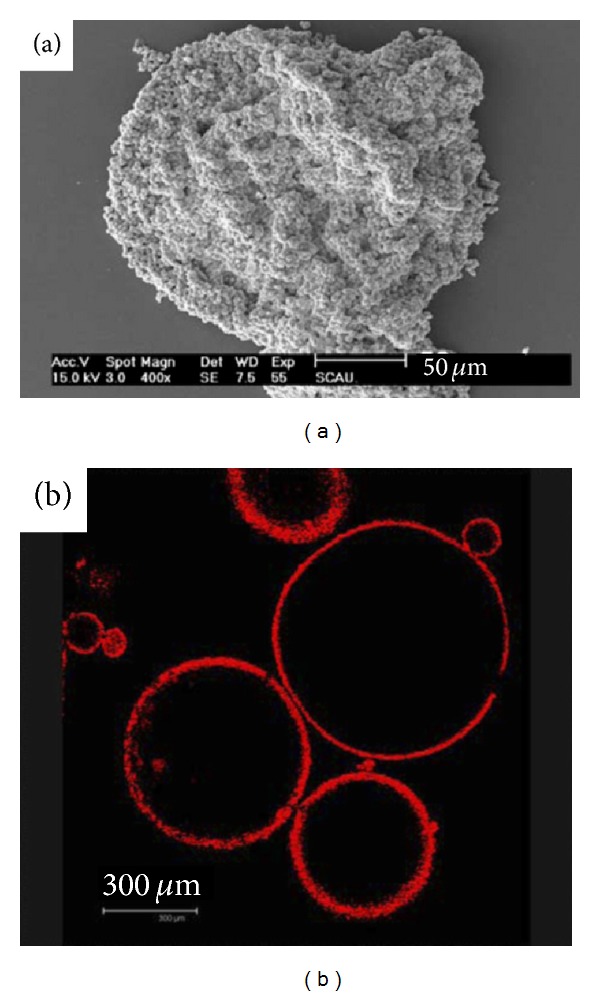
Morphology of the colloidosomes. (a) SEM and (b) confocal laser scanning microscopy; CaCO_3_ microparticles were modified with rhodamine isothiocyanate for red fluorescence visualization. Reproduced from [114] with permission of Elsevier.

Embedding of ALG microparticles within monolithic structures such as hydrogels has been also assessed to reduce the burst effect. For example, Zhu et al. encapsulated the natural drug berberine hydrochloride within ALG microspheres using an emulsification/gelation method and then entrapped them into carboxymethyl chitosan hydrogels to produce a new drug delivery composite system [113]; particles increased the hydrogel compression strength. Another interesting strategy was the production of core-shell ALG microbeads coated with self-assembled porous CaCO_3_ microparticles (Figure 10) [114]. This novel drug delivery system was named colloidosome and it reduced the release rate of a water-soluble model drug, brilliant blue, due to the generation of a dense superficial layer of a ceramic material. The same group developed colloidosomes formed by an ALG core and a Fe_2_O_3_ shell [115].

The oral administration of peptides and proteins without detrimental effects due to fast release and degradation of the payload in the aggressive gastrointestinal fluids is one of the challenges of contemporary pharmaceutical development. Their encapsulation usually demands more intense studies owing to the fact that production processes need to be mild and maintain their structure and function unaltered. Also, compositions are more complex and usually incorporate various polymers to enhance the encapsulation efficiency. Yu et al. reported on composite pH-sensitive microparticles of ALG, chitosan, and pectin for the encapsulation of bovine serum albumin as a model protein [116]. The method was shredding and combined tripolyphosphate crosslinking of chitosan, electrostatic complexation by ALG and/or pectin with chitosan, and ionotropic gelation of ALG with calcium ions. The release at pH values of 1.2 and 5.0 was substantially slower than at 7.4, supporting the potential of this system for specific oral delivery. In another work, Chen and coworkers developed novel poly(L-histidine)-chitosan/alginate complex microcapsules for the encapsulation of haemoglobin as model protein [117]. Microparticles were spherical and with narrow size distribution and smooth surface. The encapsulation efficiency was above 85% and the protein payload as high as 40–48%. In addition, the release followed first-order kinetics with released amounts between 72% and 87% after 72 h in PBS of pH 6.8. The main parameter affecting the performance of the microparticles was the molecular weight of chitosan that was used in a concentration of 0.05%. To improve the deliverability of *α*-interferon by the oral route without degradation, Saez et al. microencapsulated it within ALG microspheres [118]; interferons are usually injected and this approach would represent a breakthrough in the immunotherapy with this immunomodulatory, antiproliferative, and antiviral agent.

**Figure 11:**
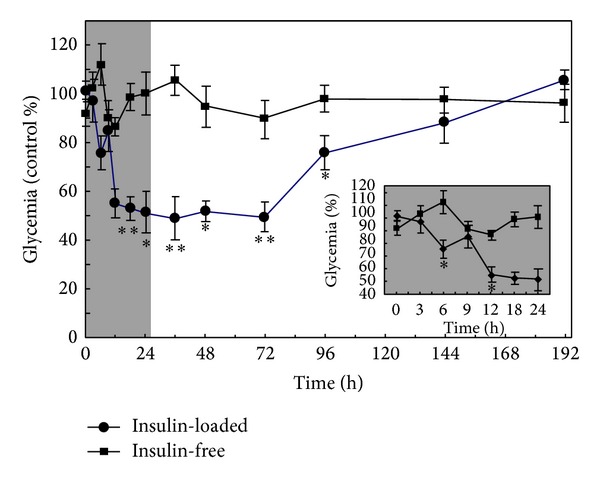
Serum glucose concentration after oral administration of insulin-free and insulin-loaded (100 IU/kg) ALG-chitosan microspheres to streptozotocin-induced diabetic rats. Statistically significant difference from insulin-free microspheres: *P < 0.05 and **P < 0.01. Reproduced from [124] with permission of Elsevier.

**Figure 12:**
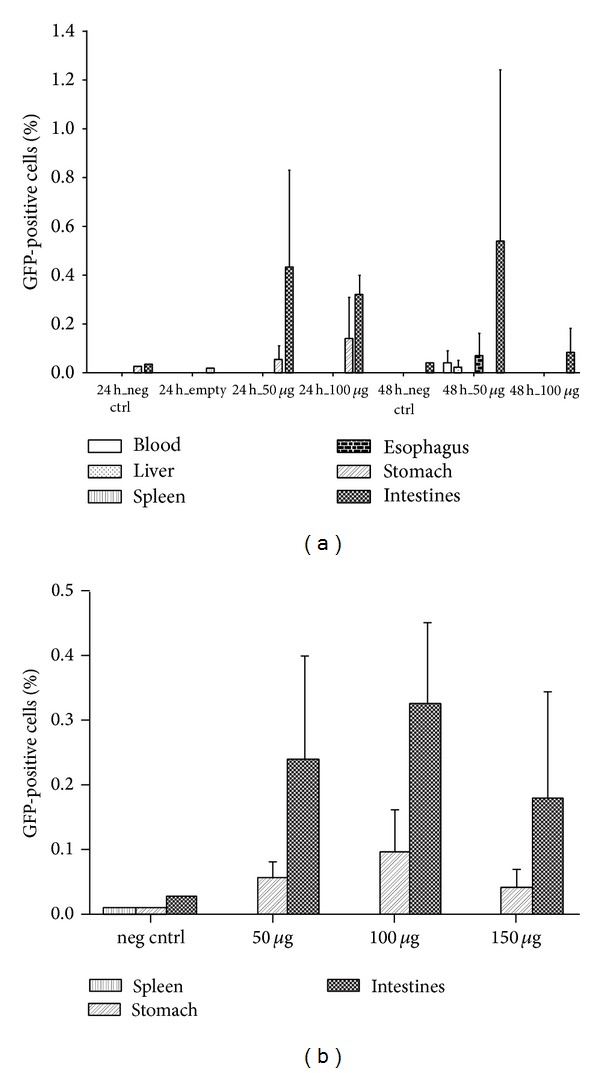
Oral delivery of pDNA with GFP reporter in ALG microspheres in mice. (a) Flow cytometric analyses showing biodistribution reflected through %GFP-positive cells among various organs 24 and 48 h after the administration of 50 *μ*g and 100 *μ*g pDNA dose. Number of animals for each dose group = 3; total number of mice = 15. (b) Dose-response assessment showing %GFP-positive cells among various mice organs 24 h after the administration of 50 *μ*g, 100 *μ*g, and 150 *μ*g dose. Number of animals for each dose group = 6 (except for 150 *μ*g-dose group which had only 3); total number of mice = 18. All measurements were done in triplicates. Results represent mean ± standard error with the basal levels of expression from negative controls subtracted from expression levels observed in dosed groups. Reproduced from [130] with permission of Elsevier.

Due to the chronic nature of the treatment of diabetes that entails painful and stressful frequent injections and the great morbidity of the disease in the developed world, insulin appears as one of the most appealing proteins to investigate novel drug delivery systems of administration by nonparenteral routes. In this context, Neufeld and collaborators have devoted efforts to develop different platforms for oral insulin [30, 119–122]. Employing an emulsion/internal gelation method, they produced small insulin-loaded ALG microparticles (<10 *μ*m) with high encapsulation efficiency [30]. To improve the recovery, a centrifugation and dehydration process was implemented. The stability of insulin in the gastric medium was also increased by reinforcing ALG microparticles with chitosan and dextran sulfate [123]. A similar concept was followed by Zhang et al. that also assessed glycemia levels in streptozotocin-induced diabetic rats with promising results (Figure 11) [124]. Glucose levels were maintained at a minimum level over 72 h as opposed to free insulin. The maintenance of constant low glucose plasma concentrations is critical to prevent the development of complications and the organic deterioration associated with the disease [125]. Another approach was the reencapsulation of bovine insulin-loaded ALG particles within poly(lactic-*co*-glycolic) acid microparticles [126]. Composite microparticles showed a diameter of approximately 22 *μ*m and a porous surface and they sustained the release over 130 days. Thus, these systems are more promising for parenteral than for oral administration. Other proteins that have been encapsulated within ALG microparticles include the proteases papain [127] and subtilisin [128]. More recent works have also explored the encapsulation of other sensitive biologicals such as adenoviruses [129] and plasmid DNA (pDNA) [130], though their application is not always envisioned for oral administration. For example, Nograles et al. encapsulated a mammalian expression vector bearing a green fluorescent protein (GFP) reporter gene within ALG microparticles with a diameter of 40–60 *μ*m and administered them to mice. Green fluorescence was detected in intestinal cells (Figure 12) [130]. While ALG has been mainly used as the major component of the microparticle matrix, other studies assessed the performance of ALG as a coating agent to protect and sustain the release of encapsulated payloads and to confer mucoadhesiveness. The protective nature of ALG in the stomach stems from its reduced solubility at low pH values due to the protonation of the pendant carboxylic moieties [21, 33, 131]. For example, Li et al. developed bovine serum albumin-loaded chitosan microparticles and coated them with ALG using the layer-by-layer method [132]. This modification significantly reduced the burst effect and prevented the degradation of the model protein in 0.01 M HCl for 2 h, suggesting that it would stabilize it during the gastric transit.

**Figure 13:**
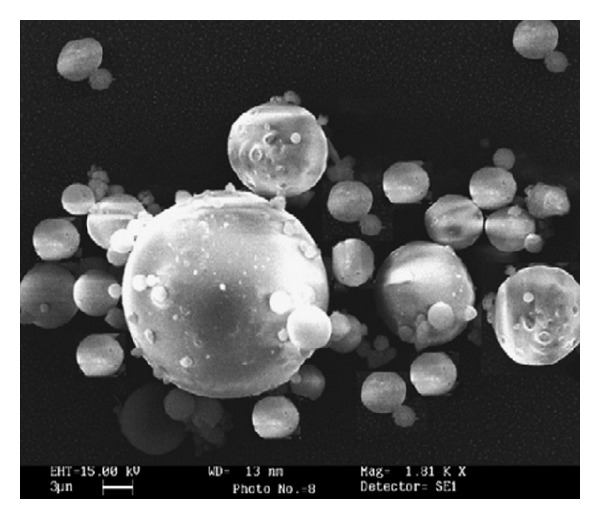
Isoniazid-loaded ALG microparticles obtained by a simple emulsion method. Reproduced from [133] with permission of Elsevier.

Another advantage of ALG is related to its ability to efficiently encapsulate hydrophilic and hydrophobic drugs [133–138]. It should be stressed though that in the case of hydrophilic drugs, ionotropic gelation is not feasible due to very low encapsulation efficiency that stems from the fast escape of the drug to the crosslinking medium. In this context, the methodology needs to be conveniently modified. Rastogi et al. studied the encapsulation of isoniazid, a first-line antituberculosis water-soluble drug, for enteric release employing an emulsion technique [133]. On the one hand, the encapsulation efficiency was 91%. On the other, the relatively high viscosity of ALG solutions jeopardized the production of products with narrow size distribution (Figure 13) [133]. This is a drawback when compared to the ability of more complex techniques to render more uniform size populations. Conversely, hydrophobic drugs can be incorporated in the form of small crystals homogeneously dispersed in the ALG matrix, as reported for the hypoglycemic drug gliclazide [134] and the anti-inflammatory prednisolone [138], or encapsulated within nanoparticles of a hydrophobic polymer (e.g., poly(lactide)) and then reencapsulated within ALG, as shown for silymarin [137]. The dispersion of poorly water-soluble drugs (e.g., nateglinide) in ALG using organic solvents has been also pursued, though the fast release is probably anticipated [139].

ALG encapsulation could be also exploited to improve the pharmacokinetics of drugs displaying a relatively short half-life. For example, Abdelbary et al. encapsulated the antidiabetic drug glipizide within pure ALG microparticles and combinations with chitosan or poly(acrylic acid) [140]. Due to a sustained release, the *t*_1/2_ was extended from 4 to 9 h.

The good cytocompatibility of ALG has been also assessed for the encapsulation of probiotic bacteria (e.g.,* Lactobacillus acydophilus*,* Bifidobacterium lactis*) that are sensitive to gastric fluids [141]. Incorporation of xanthan gum (0.5%) as a hydrophilic retardant polymer or cellulose acetate phthalate (1%) as a gastroresistant polymer to 3% ALG microparticles increased bacterial survival from 63% to 76%. Moreover, when the standard production technique was replaced by an atomization one, the size of the particles was reduced and the survival increased up to 91% after 24 h in gastric-like medium.

Other works exploited the properties of ALG matrices to develop novel biosensing, imaging, and diagnosis tools, though these developments are mainly envisioned as external or implantable devices and not for oral administration [142–144]. Following the revolution in therapeutics led by nanotechnology, the production of ALG nanoparticles for oral drug administration has also emerged as an appealing technology platform. The following section will address this specific research avenue.

### 3.2. Alginate Nanoparticles

**Figure 14:**
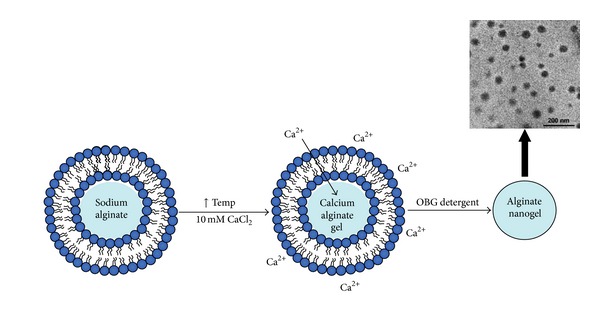
Production of ALG nanoparticles using liposomal templates. Reproduced and adapted from [156] with permission of the American Chemical Society.

**Figure 15:**
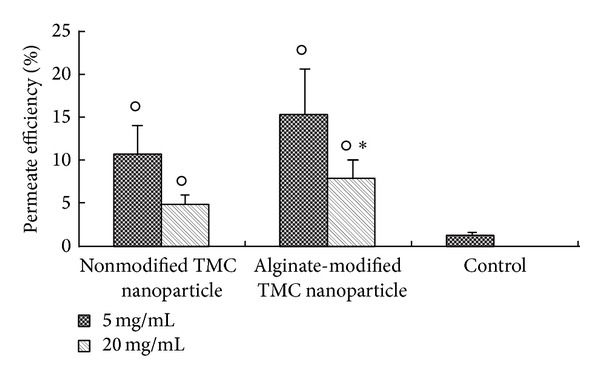
Observed permeate efficiency of bovine serum albumin-loaded TMC nanoparticles, ALG-modified TMC nanoparticles at low (5 mg/mL) and high concentration (20 mg/mL), and control (bovine serum albumin solution) using Caco-2 cell monolayer. Circles indicated permeate efficiency that was significantly higher (*P* < 0.01) than that of the control. Asterisks indicated permeate efficiency that was significantly higher (*P* < 0.05) than that of nonmodified TMC nanoparticles. No significant difference was observed between nonmodified and ALG-modified TMC nanoparticles at 5 mg/mL. All data are mean ± S.D. (*n* = 4). Reproduced from [168] with permission of Elsevier.

Nanotechnology has opened new possibilities to control and manipulate the matter. From a biomedical perspective, it enabled capitalization on novel properties of biomaterials to enhance the prevention, diagnosis, and treatment of disease and led to the emergence of the new field of theranostics [10, 11, 145]. Based on the successful experience of ALG microparticles, the study of ALG nanoparticles was expected to gain impulse due to the benefit of particle size reduction to reach cellular and subcellular structures in the gastrointestinal epithelium and subepithelial dome regions that are relevant for mucosal vaccination [146] and drug transport to the blood stream [147]. However, as opposed to chitosan, another mucoadhesive polysaccharide that has been mainly used to develop nanoparticles, the literature on ALG nanoparticles is relatively scarce. One of the challenges was the modification of production methods to achieve such small sizes. For example, the conventional ionotropic crosslinking with calcium ions was usually modified [148, 149] or replaced by water-in-oil nanoemulsions [150, 151] and polyelectrolyte approaches with chitosan [152, 153]. To overcome this, researchers also used other nanocarriers as templates (e.g., chitosan nanoparticles, liposomes) that were further surface-modified with ALG to confer mucoadhesiveness [154, 155]. For example, Haidar and coworkers produced core-shell hybrid nanoparticles by the layer-by-layer assembly of ALG and chitosan on liposomes for the delivery of a growth factor [155]. Conversely, Hong and collaborators used the core of liposomes with a high bilayer melting temperature as reaction vessels to template the assembly of ALG [156]. In this context, ALG was encapsulated in the liposomal core and exposed to a calcium chloride solution at a temperature above the melting point of the bilayer. This enables the passage of calcium ions into the core and the gradual gelation of ALG. Then, the liposome was removed with surfactants to give place to 120–200 nm nanoparticles ALG. The process is presented in Figure 14 [156]. As mentioned above, pure drug crystals could also be used to assemble polyelectrolyte layers. Following this rationale, artemisinin nanocrystals (766 nm) were coated with chitosan, gelatin, and ALG [157]. Following the trend presented with microparticles, a major part of the research at the interface of nanotechnology and ALG for oral administration was devoted to insulin [158–160]. At the same time, some isolated studies explored this platform for the encapsulation of other drugs [161, 162] and antigens [163]. Ahmad et al. encapsulated the antifungal drugs clotrimazole and econazole and the antituberculosis drugs rifampicin, ethambutol, isoniazid, and pyrazinamide within ALG nanoparticles [149, 164, 165] by means of a modified cation induced controlled gelification [149, 166]. After oral administration, free drugs were detectable for only 6–24 h, while the encapsulated ones between 8 and 15 days. Moreover, eight doses of econazole-loaded nanoparticles had an antibacterial effect similar effect as 112 twice-a-day doses of the free drug [165]. The findings confirmed the ability of ALG nanoparticles to cross the intestinal barrier and reach the blood stream, as opposed to microparticles that are mainly retained in the gut mucosa. This beneficial effect was also investigated to develop mucopenetrating ALG/chitosan nanoparticles for the release of amoxicillin in the treatment of the infection by* Helicobacter pylori*, a pathogen that colonizes the deep gastric mucosa lining [166]. Results indicated lower mucoadhesiveness for the combination, though greater mucopenetration, than pure chitosan. These observations were in agreement with a study by Chen et al. that showed the enhanced permeation of bovine serum albumin-loaded* N*-trimethyl chitosan (TMC) nanoparticles across Caco2 cell monolayers, when modified with ALG (Figure 15) [167]. This improvement would stem from an increased transcellular pathway, while the paracellular one remained unaltered [168]. Another mechanism behind this phenomenon would be the active uptake of these nanoparticles by Peyer's patches [168].

Aiming to develop an oral vaccine against schistosomiasis, a neglected parasitic disease, the Smrho protein was encapsulated within chitosan nanoparticles coated with ALG [169]. Due to its good chemical flexibility, some works addressed the chemical modification of ALG to confer gene transfection capacity [150, 170] or active targeting properties [168]. In this context, ALG nanoparticles loaded with the fluorescent probe protoporphyrin IX were modified with 5-aminolevulinic acid, a ligand that is selectively recognized by cancerous cells that over-express the folic acid receptor, to confer diagnostic capability by endoscopy [168]. Nanoparticles were endocytosed by colorectal cancer cells and the probe was released to the intracellular space and accumulated for sensitive photodynamic detection. These results, together with previous evidence of the performance of ALG for drug encapsulation and release support it as a valuable tool for the design of more complex systems in the emerging field of theranostics. At the same time, further studies will need to be addressed to capitalize on this potential and extend the applications of ALG.

## 4. Conclusions and Perspectives

Owing to very good biocompatibility and approval by the US-FDA as food additive, ALG has gained a preferential place among pharmaceutical excipients for the development of advanced drug delivery systems for mucosal administration. The greater interest of the scientific community in ALG coincided with the revolution in therapeutics led by nanomedicine. This together with a great versatility to conduct fine chemical modifications and to tailor specific features by combining it with other natural and synthetic polymers have sustained the steady increase of the research at the interface of ALG and microparticles and nanoparticles during the last decade. At the same time, it should be stressed that strict isolation and purification protocols are demanded to ensure low concentrations of endotoxins and immunogenic residues and the interbatch reproducibility. These issues are even more critical to extend the use of ALG to parenteral administration routes. The present spotlight review overviewed the most recent works of ALG as a technology platform to develop microparticles and nanoparticles for oral drug delivery. The most outstanding features are mucoadhesiveness and mucopenetration that increase the passage of drug payloads through the gastrointestinal epithelium. This could be capitalized to enhance local and systemic delivery, increase oral bioavailability, and prolong release. Research on ALG microparticles has been more profuse with a broad spectrum of production methods that range from simple ionotropic gelation to more complex equipment to control size and size distribution and to ensure reproducibility and scale up. Conversely, the research of ALG nanoparticles has been limited to a few payloads, insulin being the most intensively assessed. In any event, ALG bears a great potential and its more extensive implementation in the development of innovative nanodrug delivery systems with translation potential is a matter of time.
